# Association Between Use of a Primary Care Health Check-Up Tool and the Early Detection of Chronic Diseases: A Non-Randomised Comparative Study from the POZ PLUS Pilot Programme in Poland

**DOI:** 10.3390/medicina62010056

**Published:** 2025-12-28

**Authors:** Daria Małecka, Anna Tyrańska-Fobke, Katarzyna B. Kubiak, Aleksandra Kuich, Andrzej Zapaśnik, Marlena Robakowska

**Affiliations:** 1 BaltiMed Gdansk Clinic, 80-041 Gdańsk, Poland; 2 Department of Public Health & Social Medicine, Medical University of Gdańsk, 80-210 Gdańsk, Polandmarlena.robakowska@gumed.edu.pl (M.R.)

**Keywords:** chronic diseases, prevention, pilot program

## Abstract

*Background and Objectives*: Early detection of chronic diseases is essential for improving health outcomes and reducing long-term complications. In Poland, the POZ PLUS pilot programme introduced the Health Check-up (Bilans Zdrowia, BZ) tool, a structured preventive assessment designed to support early identification of chronic conditions in primary care. This study aimed to assess the association between participation in the Health Check-up and the detection (diagnostic yield) of hypertension, type 2 diabetes, lipid metabolism disorders, elevated fasting blood glucose, hypothyroidism, and non-toxic goiter by comparing outcomes in an intervention group and a control group. *Materials and Methods*: A non-randomised comparative study was conducted using routine clinical data from Health Check-ups performed within the POZ PLUS pilot. Detection rates were compared with those obtained in standard primary care practice during the same period. The study group consisted of 865 adults who met the inclusion criteria and underwent the BZ procedure, while the control group comprised 3199 patients with comparable eligibility who received usual care. Data analysis was performed using R and RStudio. *Results:* Hypertension detection was similar in both groups: 4.6% (95% CI: 3.3–6.3%) in the intervention group versus 4.5% (95% CI: 3.8–5.3%) in the control group (*p* = 0.9505). No significant difference was observed in type 2 diabetes detection: 0.7% (95% CI: 0.3–1.5%) versus 0.4% (95% CI: 0.2–0.7%) (*p* = 0.4134). In contrast, detection rates were significantly higher in the Health Check-up group for lipid metabolism disorders (10.3% vs. 2.6%; *p* < 0.001), abnormal fasting glucose (2.9% vs. 1.8%; *p* = 0.0465), and thyroid diseases, including hypothyroidism and non-toxic goiter (4.3% vs. 2.3%; *p* = 0.0016). *Conclusions:* The Health Check-up tool was associated with higher detection rates of lipid disorders, impaired fasting glucose, and thyroid diseases compared with usual care, suggesting increased diagnostic yield under a structured preventive assessment pathway. Further research should evaluate downstream clinical outcomes and cost-effectiveness. Given the non-randomised design and differential diagnostic intensity between groups, these findings should be interpreted as associations with diagnostic yield rather than causal effects on disease incidence or clinical outcomes.

## 1. Introduction

Currently, there is a noticeable increase in the average life expectancy of the population worldwide. This is due, among other things, to continuous advances in medicine and medical science, as well as a significant improvement in living conditions in highly developed countries. As a result of these conditions, mortality from acute diseases is decreasing, and chronic diseases have become one of the most common causes of death worldwide [1].

According to data from the Global Burden of Disease Study 2021, non-communicable diseases accounted for 75%, or 43 million, of non-pandemic-related deaths. The most common causes were cardiovascular diseases (19 million), followed by cancer (10 million), chronic respiratory diseases (4 million), and diabetes (over 2 million) [2]. In Poland, according to data provided by the Central Statistical Office, the main causes of death in 2023 were cardiovascular diseases, accounting for 37% of all deaths [3]. Although the rate of deaths that could have been prevented by preventive measures in Poland has fallen over the last decade, it remains higher than the European Union average [4]. In 2020, the age-standardised mortality rate per 100,000 people in Poland was 275, while in most EU countries it was 180. The main factors contributing to higher rates in Poland include the most common behavioural risk factors, such as smoking, poor eating habits, and excessive alcohol consumption [5]. To counteract these problems, preventive interventions should become a key element in the organization of healthcare. Their main goal should be to support and maintain health, minimize risk factors, detect diseases early, and prevent their complications [6]. Therefore, properly designed and regularly performed preventive health checks can be crucial for modern preventive medicine and be the future of prevention [7]. Primary health care (PHC) plays a significant role in the prevention, early detection, and management of chronic diseases [8].

In response to this, a pilot model of POZ PLUS was implemented in Poland, which lasted from 1 July 2018, to 30 September 2021. One of the elements implemented as part of this model was the Health Check-up (Bilans Zdrowia, BZ), which was designed as a set of standardized tests aimed at preventing or early detection of chronic diseases. In the facility under study, this tool was partially modified by the authors in order to adapt it to the conditions of the facility and improve its practical applicability. The main objective of the BZ was to stratify the population by identifying healthy patients and potentially ill patients, which would enable the management of the type and number of services tailored to the needs of a given group of patients in the future [9].

The age range of 20–65 years included in the Health Check-up was defined centrally as part of the national POZ PLUS pilot programme and applied uniformly across all participating primary care facilities. This design reflected the programme’s broad preventive objective, which extended beyond cardiovascular risk assessment to include early detection of metabolic and endocrine disorders that may also affect younger adults. As mentioned, the assessments covered populations aged 20 to 65 and were directed at patients for whom the healthcare provider did not have complete or up-to-date medical data on their health status. In addition, patients recruited to the program in the last 12 months should not have been hospitalized and should not have undergone diagnostic tests that overlapped with the check-up tests, nor should they have received services from a specialist (AOS) or primary care physician (POZ) for the following chronic diseases: diabetes (E10, E11), hypertension (I10–I15), ischemic heart disease (I20–I25), other chronic heart diseases (I42–I52), consequences of cerebrovascular diseases (I69), atherosclerosis and other arterial diseases (I70–I73), chronic renal failure (N18), chronic liver diseases (B18, K70, K72–K74), malignant neoplasms (C00–C97), inflammatory arthropathies (M05–M09), and systemic connective tissue diseases (M30–M36) [10].

Participation in the POZ PLUS pilot programme was voluntary and preceded by written informed consent. Initial contact with potential participants was established through routine interactions within the primary care practice. Health Check-ups were offered to patients registered on the active primary care list of the practice for whom the available medical documentation did not include complete or up-to-date information on health status.

Patients were most commonly informed about the Health Check-up during routine primary care visits, typically through direct physician–patient communication. Additional recruitment pathways included information provided at the time of appointment registration by administrative staff. To support targeted telephone outreach, programme coordinators used internally prepared practice-level reports generated from the electronic health record system to identify patients potentially eligible for the Health Check-up, for example, based on age criteria and limited recent contact with the practice, indicating a lack of current health information in the medical records. Programme coordinators then contacted these patients by telephone to inform them about the possibility of participation. During such a conversation, the patient received basic, general information about how the BZ works, along with questions about the patient’s medical history, including chronic diseases, hospitalizations, preventive examinations, medications taken, etc., for which they should be prepared. The BZ began during the patient’s first visit to the facility after qualifying for the check-up. The patient provided material for the tests that were necessary for the basic check-up, i.e., complete blood count, ESR, blood glucose, lipid profile (including total cholesterol, LDL cholesterol, HDL cholesterol, and triglycerides), and urinalysis. During this visit, the coordinator began filling out the check-up questionnaire with the patient. The check-up questionnaire included questions about the patient’s personal data, followed by a subjective assessment of their general health, including physical activity, and family history, including cancer and chronic diseases. This was followed by an extensive part of the further interview and physical examination, which included questions about the patient’s current and past medical history, participation in screening and preventive programs, along with the date and results of the tests. This was followed by an interview about stimulants, pharmacotherapy, vaccinations, and treatment outside of primary health care (procedures, surgeries, hospitalizations, AOS care), followed by the patient’s reported complaints. At the end of this part of the questionnaire, anthropometric measurements, blood pressure and heart rate measurements were taken, and questions about mental well-being were asked [10].

The next stage began with a medical examination, starting with the physical examination section. The rest of the questionnaire was completed by the doctor, based on the continuation of the medical interview with the patient, the assessment of the anthropometric examination, and the results of laboratory tests. The doctor then decided in each case whether the patient should be referred for an in-depth check-up or whether the basic check-up should be completed. A referral for an in-depth check-up was indicated when the doctor, based on the data collected during the medical interview, noted risk factors that could predispose the patient to the disease. Finally, the necessary referrals for tests were issued, and then it was the coordinator’s task to arrange another appointment to conclude the check-up based on the types of tests ordered by the doctor.

At the final visit concluding the check-up, the doctor assessed the patient’s health, taking into account the risk of chronic disease, and classified them into one of the following groups depending on their health status: healthy patient–no risk factors; healthy patient–no symptoms but with risk factors; patient with suspected chronic disease, chronically ill patient–currently without symptoms, stable; chronically ill patient–with current symptoms, requiring stabilization [9]. The National Health Fund (NFZ) provided for four groups, but as a result of one of the modifications to the questionnaire, an additional health status category was added at the facility, covering patients with suspected chronic disease. The performance and completion of the comprehensive or basic BZ ended with the preparation of an Individual Health Management Plan (IHMP) for each patient on the basis of the BZ questionnaire. The IHMP had to include the results of the tests carried out as part of the program and medical recommendations [10]. The questionnaire on the patient’s health status included information on further treatment. During the final check-up visit, the doctor could refer the patient for further tests outside the check-up package, refer them to a specialist, or issue a card for oncological diagnosis and treatment (DILO) or a preventive program (prevention of cardiovascular disease, chronic obstructive pulmonary disease, breast cancer, cervical cancer, screening for colorectal cancer). In addition, the patient could be referred for educational visits, specialist dietary consultations, or specialist psychological consultations [9].

The aim of the presented study was to evaluate the effectiveness of the BZ tool in detecting. The aim of the present study was to compare detection rates (diagnostic yield) of selected disease entities, defined according to the International Classification of Diseases, 10th Revision (ICD-10)—essential hypertension (I10), type 2 diabetes mellitus (E11), lipid metabolism disorders (E78), elevated blood glucose (R73), hypothyroidism (E03), and non-toxic goiter (E04)—between patients assessed using the Health Check-up (BZ) tool and those receiving usual primary care in the study and control groups of patients aged 20–65 years covered by primary health care in a provincial city in Poland.

## 2. Materials and Methods

The BZ tool was piloted as part of the POZ PLUS program among 1303 patients at the BaltiMed primary care facility in Gdańsk, located in the Pomeranian Province in northern Poland. This facility, on the basis of agreements with the National Health Fund, provides primary and outpatient specialist healthcare to patients. The facility provides medical care for over 10,000 patients in primary health care, within which 43,777 consultations were provided in 2024 and 19,395 in outpatient specialist care. All data and patients included in the analysis come exclusively from this facility, which was one of 47 in Poland to implement the POZ PLUS pilot program [11].

A non-randomised comparative retrospective study was conducted using routinely collected electronic health record (EHR) data from primary care, comparing detection of selected newly diagnosed conditions between patients who completed BZ as part of the POZ PLUS pilot programme and eligible patients receiving usual primary care during the same period (1 July 2018–31 October 2021). The data for the study were extracted from the medical records of patients included in the study, which are stored in the Serum program and are at the disposal of the BaltiMed clinic.

The sample consisted of patients belonging to the study group, i.e., those who underwent the BZ procedure, and the control group, which consisted of patients who met the criteria for inclusion in BZ but did not participate in the procedure.

The control group was identified from the same primary care practice EHR and consisted of patients aged 20–65 years who did not participate in the Health Check-up during the study period but met the programme-level eligibility criteria. Eligibility was operationalised by confirming the absence of prior ICD-10 diagnoses corresponding to the analysed disease entities before the index date. Due to the retrospective nature of the study, some programme criteria (e.g., recent hospitalisation or overlapping diagnostic testing) could not be verified with the same precision for non-participants as for BZ participants.

Patients who met the following criteria were included in the study: participation in the BZ procedure, age between 20 and 65 years, and no previous diagnosis of the indicated disease entities: I10, E11, E78, R73, E03, E04 before undergoing the check-up. The exclusion criteria were: no participation in the BZ procedure, diagnosis of at least one of the examined disease entities before the start of the BZ procedure. The inclusion criteria for the control group were: no participation in the BZ procedure, age between 20 and 65, no diagnosed disease entities: I10, E11, E78, R73, E03, E04 before the start of the Health Check-up programme at the facility. The exclusion criteria for this group were: age below 20 or above 65, diagnosed disease entities: I10, E11, E78, R73, E03, E04 before the start of the checkups.

The qualification process provided for the possibility of exceptions, which allowed the doctor to make an individual decision to include a patient in the program. Such situations included cases where the aim was to expand the diagnosis towards multimorbidity. However, patients included in the Health Check-up under such exceptional circumstances were not eligible for the present analysis and were excluded at the stage of study group selection to preserve comparability with standard eligibility criteria.

It should be noted that eligibility for BZ was defined at the programme level and required the absence of prior contact with primary or specialist care for a broad range of chronic diseases, including all hypertensive disease categories (ICD-10 codes I10–I15). Consequently, the study population had already been preselected to exclude individuals with any previously diagnosed form of hypertension. Therefore, in the present analysis, ICD-10 code I10 (essential (primary) hypertension) was used as the endpoint to identify newly detected cases at the time of first diagnosis during the Health Check-up, while no additional exclusion based on codes I11–I15 was required at the analytical stage.

Study outcomes were defined as newly established ICD-10 diagnoses recorded in the electronic health record by primary care physicians. Diagnostic decisions were made as part of routine clinical practice, in accordance with prevailing national and international clinical guidelines, and were based on clinical assessment and confirmatory testing when indicated. Due to the retrospective use of routinely collected EHR data, detailed information on specific diagnostic thresholds (e.g., number of measurements or laboratory cut-offs and repeat testing requirements) was not consistently available for all patients and could not be uniformly reconstructed.

Because the BZ assessment included systematic laboratory testing and structured evaluation, whereas usual care was opportunistic, differential diagnostic intensity between groups was expected and may increase detection of subclinical abnormalities in the BZ group.

In cases where a new ICD-10 diagnosis was recorded during the Health Check-up, the medical records were reviewed to confirm that the condition had not been previously recorded or treated within the facility. Information on care outside the study facility (e.g., private healthcare) was considered only when documented in the EHR (e.g., patient-reported history or transferred medical documentation). Only diagnoses considered newly established based on available documentation were included in the analysis.

For patients in the Health Check-up (BZ) group, the index date was defined as the date of the first encounter initiating the BZ assessment (i.e., the visit at which the check-up process started and laboratory testing and questionnaire assessment were undertaken). For control patients, the index date was defined as the first primary care encounter recorded during the pilot period (1 July 2018–31 October 2021) at which the patient met the operationalised eligibility criterion (i.e., no prior ICD-10 diagnoses corresponding to the analysed entities recorded before that date) and did not undergo the BZ assessment. Outcomes were ascertained as newly recorded ICD-10 diagnoses occurring after the index date until 31 October 2021. This uniform observation end date was applied to both groups to allow capture of diagnoses resulting from investigations initiated near the end of the pilot period (e.g., laboratory tests ordered during late Health Check-up visits). Therefore, estimates represent cumulative detection proportions rather than incidence rates based on person-time.

The analysis was based on routinely collected EHR data. Information on age and sex was available for all patients. Other potential confounders, such as BMI, smoking status, lifestyle factors, or detailed measures of prior healthcare utilisation, were not consistently or systematically recorded for both groups during the study period and therefore could not be reliably included in the analysis.

Based on the data from both groups, the detection rate was calculated, defined as the ratio of the number of newly detected cases of hypertension and type II diabetes, lipid metabolism disorders, elevated blood glucose, hypothyroidism, and non-toxic goiter, to the total number of patients in each study group.

95% confidence intervals for the detection rate were determined. The detection rates in the BZ group and in the control group for individual diagnoses/diseases were compared using a two-sample test for equality of proportions with Yates’ continuity correction. A significance level of 0.05 was adopted. Data analysis was performed using R (version 4.4.1) and RStudio (version 2024.12.0).

The study did not require additional patient consent for the use of data. The data were anonymised. The study was approved by the Independent Bioethics Committee at the Medical University of Gdańsk, registered under number KB/240/2024 on 17 May 2024.

## 3. Results

### 3.1. Characteristics of the Patients Included in the Study

A total of 4064 patients were analysed in the study. Of the 1303 patients who underwent intervention as part of the Health Check-up, 865 people who met the inclusion criteria were qualified for the study group, including 521 women and 344 men. The control group included 3199 patients, including 1577 women and 1622 men.

Detailed characteristics of the patients are presented in Table 1.

In the study group, women accounted for 60.2% (*n* = 521) and men for 39.8% (*n* = 344), while in the control group, women accounted for 49.3% (*n* = 1577) and men for 50.7% (*n* = 1622). The study group had a higher average age of 44 years, compared to 39 years in the control group. A similar difference was also observed in the median age (44 years and 38 years).

### 3.2. Frequency of Detection of Selected Diseases

Table 2 presents data on the frequency of detection of selected ICD-10 diagnoses among patients after Health Check-up and the control group.

The analysis of the frequency of detection of selected ICD-10 diagnoses showed varied results.

Hypertension (I10) occurred with a similar frequency of detection in both groups: 4.6% (95% CI: 3.3–6.3%) in the study group and 4.5% (95% CI: 3.8–5.3%) in the control group (*p* = 0.9505). The *p*-value does not indicate a significant difference between the group of patients after the intervention and the control group; however, the small number of newly detected cases limits the precision of the estimates.

For type II diabetes (E11), the detection rate in the study group was 0.7% (95% CI: 0.3–1.5%), and in the control group it was 0.4% (95% CI: 0.2–0.7%) (*p* = 0.4134). The *p*-value did not indicate a statistically significant difference between the groups; however, the relatively small number of newly detected cases may have limited the precision of the estimates.

A significantly higher frequency was found in the case of early diagnosis of lipoprotein metabolism disorders (E78)–10.3% (95% CI: 8.3–12.7%) in the group after the examination, compared to 2.6% (95% CI: 2.1–3.2%) in the control group (*p* < 0.001). The recorded *p*-value confirms the existence of a statistically significant difference between the group after BZ and the control group.

Abnormal fasting blood glucose (R73) was diagnosed more frequently in patients after the examination at 2.9% (95% CI: 1.9–4.3%) than in the control group at 1.8% (95% CI: 1.3–2.3%), respectively (*p* = 0.0465). The *p*-value in the analysis of the frequency of detecting abnormal fasting blood glucose (R73) may indicate a significant difference between the study groups.

**Figure 1:**
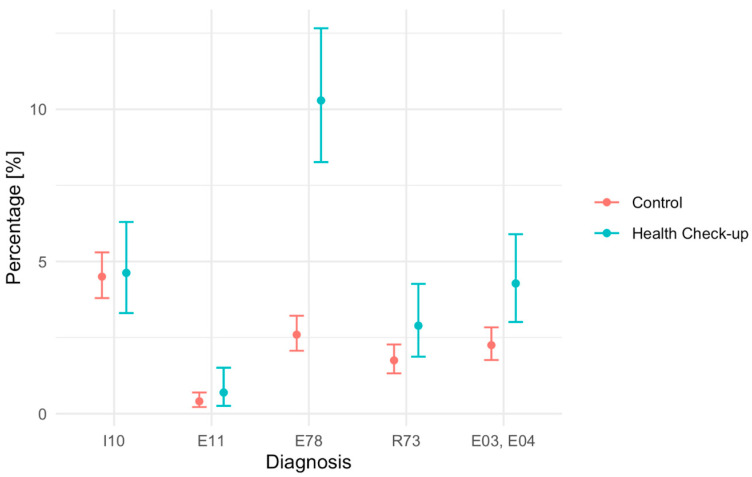
Percentage of diagnoses with a 95% confidence interval.

In the case of hypothyroidism and nodular goiter (E03, E04), a higher incidence was observed in the study group: 4.3% (95% CI: 3.0–5.9%) than in the control group: 2.3% (95% CI: 1.8–2.8%) (*p* = 0.0016). The value obtained confirms the existence of a statistically significant difference between the intervention group and the control group. Figure 1 presents a summary of the above results.

More than one diagnosis of the analyzed disease entities was made in 32 patients after BZ and in 67 patients in the control group. The frequency of diagnosis of more than one disease entity was higher in the study group (3.7%, 95%CI: 2.63–5.18) compared to the control group (2.00%, 95% CI: 1.57–2.55) (*p* = 0.00523).

## 4. Discussion

The aim of this study was to assess the association between participation in the Health Check-up and the detection rates of selected disease entities. Our results primarily reflect differences in diagnostic yield under structured versus opportunistic assessment in routine primary care. Based on the analysis, it can be concluded that, with regard to lipoprotein metabolism disorders (E78), abnormal fasting blood glucose (R73), and thyroid diseases, i.e., hypothyroidism and nodular goiter (E03, E04), statistically significant differences were found, with a higher detection rate in the group of patients after the intervention. For the other conditions studied, the results did not show statistically significant differences between the Health Check group and the control group.

It is important to note that the present study did not assess downstream clinical processes or outcomes, such as initiation of treatment, adherence to therapy, control of identified risk factors, or long-term morbidity and mortality. Consequently, increased detection should not be interpreted as evidence of improved clinical outcomes. Whether earlier identification of abnormalities within the Health Check-up translates into meaningful health benefits requires further investigation. The observed differences should be interpreted as associations rather than causal effects, given the non-randomised design and potential for self-selection and unmeasured confounding.

### 4.1. Lipid Metabolism Disorders

Elevated blood cholesterol levels are one of the main risk factors for cardiovascular disease in Poland. They affect approximately 60% of the adult Polish population aged 18–79. A significant proportion of people with high cholesterol levels are unaware of their condition, which is usually asymptomatic in its early stages, and therefore live in the belief that they are completely healthy [12]. Regular monitoring of the lipid profile is an important element in the prevention of cardiovascular disease, as it increases the likelihood of early detection of disorders and enables the implementation of appropriate interventions. As a result, it reduces the risk of both first and recurrent cardiovascular events. Lipid disorders are among the main and, at the same time, modifiable risk factors for cardiovascular disease [13].

Many scientific studies consistently confirm a log-linear relationship between absolute changes in LDL-C cholesterol levels in the blood and the risk of cardiovascular events (ASCVD). According to the current ESC/EAS guidelines, it is recommended to take measures focused on reducing the incidence of cardiovascular disease (CVD), mainly through blood lipid control [14].

A review of the international literature indicates a variety of preventive strategies in healthcare systems for the early detection of, among other things, lipid disorders. In the United Kingdom, a population program called NHS Health Check (National Health Service Health Check) has been in place since April 2009, aimed at people aged 40–74 without previously diagnosed cardiovascular disease. Its goal is the early detection of risk factors and diseases such as hypertension, hypercholesterolemia, and type 2 diabetes through regular screening tests carried out in primary health care [15]. Many systematic reviews and meta-analyses have been conducted on the effectiveness of the program. One review of a sample of 140,899 participants showed that during the first 12 months after participating in the program, the number of new diagnoses of hypercholesterolemia was 11% higher among program participants compared to the control group [16]. Data from the Clinical Practice Research Datalink (CPRD) also suggest that new diagnoses of lipid disorders were more common among participants in the NHS Health Check program. In a study by Chang et al., a significantly higher incidence of familial hypercholesterolemia was reported in program participants compared to non-participants within two years of screening [17]. These results suggest that participation in the program may be associated with a higher likelihood of detecting previously undiagnosed lipid disorders, although this does not determine its overall clinical effectiveness [17]. Although the results of the studies are not always clear-cut, it can be assumed that participation in the NHS Health Check program is associated with increased detection of cardiovascular disease risk factors and more frequent diagnoses [18].

In comparison with population-based programmes such as the NHS Health Check, where increased detection of hypercholesterolaemia has also been reported, the detection rate observed in our study was of similar direction but occurred within a younger and broader age range. This suggests that structured preventive assessments may facilitate identification of lipid disorders in primary care settings, although differences in programme design and eligibility criteria limit direct comparability.

### 4.2. Elevated Blood Glucose Level and Type 2 Diabetes

The present study showed statistically significant differences in the prevalence of abnormal fasting blood glucose levels, while the differences in the detection of type 2 diabetes did not reach statistical significance. These results may indicate the usefulness of Health Checks in identifying patients in the early stages of metabolic risk, when effective prevention of progression to type 2 diabetes is still possible. In the context of health policy, this emphasizes the validity of implementing early detection strategies targeting prediabetes. Epidemiological data indicate the significant scale of the problem of type 2 diabetes in Poland. In 2021, the disease was diagnosed in approximately 2.67 million people, impaired glucose tolerance was diagnosed in approximately 2.45 million people, and impaired fasting glucose was diagnosed in approximately 747,700 people. Problems with glucose metabolism were identified as one of the key health issues in the Health Needs Maps for 2022–2026 [19]. In response to this concern, the Recommendation of the President of the Agency for Health Technology Assessment and Tariff System (AOTMiT) of 11 December 2024, on recommended medical technologies, activities carried out as part of health policy programs, and the conditions for implementing these programs, concerning the prevention of type 2 diabetes, confirms the validity of preventive measures for type 2 diabetes. According to the document, in Poland, it is recommended to conduct fasting glucose screening among the population over 45 years of age and among younger people who are overweight or obese and have an additional risk factor for type 2 diabetes, but also among people diagnosed with prediabetes. It was also indicated that these measures should be supplemented with interventions aimed at lifestyle modification, which corresponds to the current health and prevention needs of the population. The recommendation of the President of AOTMiT fits into the broader context of measures that should be aimed at early detection of glycemic disorders and prevention of full-blown disease, which may be of significant importance from both a clinical and systemic perspective [20,21,22].

An example of type 2 diabetes prevention activities in Poland is the “w Pomorskim nieCukrzymy” (No Diabetes in Pomerania) program implemented in the Pomeranian Province as part of the Regional Health Program. The project includes extensive preventive measures carried out in selected primary health care facilities, aimed at the early detection of type 2 diabetes and prediabetes, and the development of healthy habits among adult residents of the region. The main objective of the program is to reduce the incidence of type 2 diabetes among the inhabitants of the Pomeranian Province. The program is open to people of working age between 35 and 64 who have not previously been treated for type 2 diabetes, who have not undergone an OGTT test within one year prior to joining the program, and who have scored 12 points or more in the FINDRISK questionnaire. Qualified participants then receive a referral for an oral glucose tolerance test, and if carbohydrate metabolism disorders are confirmed, they are invited to medical appointments and a comprehensive educational program. The program aims to test at least 270,000 residents of the region using the FINDRISC test, perform 30,000 OGTT tests, and involve 5000 people in the educational cycle. The expected outcomes of the program include increased physical activity and improved eating habits in 40% of participants, as well as weight and waist circumference reduction in at least one-third of overweight or obese individuals. The program is an example of a comprehensive approach to diabetes prevention, combining elements of early diagnosis, health education, and long-term evaluation of the effects of intervention [23].

In the context of European experience, a pilot study conducted in Italy in primary health care facilities on a nationwide type 2 diabetes prevention program is of interest. As part of the study, family doctors randomly recruited a group of patients with no previously diagnosed metabolic diseases. Participants initially completed the FINDRISC (Finnish Diabetes Risk Score) questionnaire, which assesses the individual risk of developing type 2 diabetes, and those with a score exceeding 9 points were referred for fasting blood glucose testing. Of the 5928 people recruited, 2168 scored >9 on the Findrisc and were referred for testing. The results proved to be clinically significant and highly effective in identifying individuals with glucose metabolism disorders—755 subjects (34.8%) were found to have elevated blood glucose levels, while 79 individuals (3.6%) were diagnosed with previously undiagnosed type 2 diabetes.

The results of this study indicate that a two-stage screening approach may also be one of the potentially effective courses of action in the strategy for preventing type 2 diabetes [24].

A different strategy has been adopted in Australia for the diagnosis of diseases related to carbohydrate metabolism disorders. For more than 6 weeks, blood glucose levels were measured in each patient who had their blood tested at the emergency department. In addition, HbA1c levels were also measured in 1267 of the 2652 samples. Diabetes was confirmed in 487 (38.4%) patients, of whom 32.3% were newly diagnosed–the patients had not been diagnosed previously, and another 347 (27.4%) were diagnosed with prediabetes. The study indicated that the development of type 2 diabetes can be prevented if patients are provided with support in achieving lifestyle changes [25]. The above data indicate that effective screening measures can take various forms.

Data from the GENVASC (Genetics and Vascular Health Check) study, which was designed to evaluate the clinical effectiveness of the aforementioned NHS Health Check program, further indicate a type 2 diabetes detection rate of 0.9% among 27,888 people who underwent NHSHC, during a follow-up period of at least 18 months [26].

In addition, the findings of the ADDITION-Europe study indicate that the greatest health benefits of screening for type 2 diabetes result primarily from early detection of the disease and rapid implementation of treatment. Based on the published literature, the study assumed that screening could accelerate the diagnosis of diabetes by 3 or even 6 years. The authors suggest that earlier diagnosis of diabetes may have a significant impact on overall mortality and the incidence of cardiovascular events [27].

Our findings support the rationale for further evaluation of structured screening approaches for type 2 diabetes in primary care, particularly with respect to earlier identification of dysglycaemia.

### 4.3. Hypertension

In our study, hypertension showed no significant differences in the frequency of diagnoses between the study and control groups. This result may be due to the fact that in an apparently healthy population, blood pressure is usually measured during routine medical visits or periodic occupational health examinations, rather than as a result of systematic preventive measures. At this point, it is also worth noting the role of case finding in the identification of hypertension [28,29]. Although, as mentioned, the statistical analysis did not show significant differences between the study and control groups, it is possible that the comparable rate is due to the above mechanism in the control sample. This refers to the active detection of disease entities during a patient’s visit for another health problem. In the study group, the check-up procedure may promote earlier detection, even before the first symptoms that carry cardiovascular risk appear. According to data from the Global Burden of Disease Study 2021, the most common cause of death was cardiovascular disease (19 million), which, according to data from 2023 provided by the Central Statistical Office, also contributed most to mortality in Poland, accounting for 37% of all deaths [3]. According to data from the National Health Fund, 9.94 million adults in Poland suffered from hypertension in 2020. Hypertension is most common in the 55–74 age group [30].

Studies show that early detection of hypertension and the implementation of appropriate interventions, such as lifestyle changes and the introduction of pharmacological treatment, can reduce the risk of cardiovascular mortality in people with newly diagnosed hypertension. This emphasizes the importance of preventive examinations, which lead to the identification of people with high blood pressure who are unaware of their health condition [31].

An example of this is a study conducted in Finland, which aimed to analyze the relationship between hypertension (people with normal blood pressure, people with hypertension detected in screening, and people taking hypertension medication from the start of the study) and mortality over a 13-year follow-up period. The study included 2569 people with cardiovascular risk factors. Among the study group, hypertension detected in screening was diagnosed in 17% of patients, 51% were normotensive, and 32% were already taking medication at the start of the study. In individuals with hypertension detected in the screening, the risk of death from cardiovascular causes was lower compared to individuals undergoing pharmacological treatment and, interestingly, comparable to normotensive individuals. Early identification of hypertension and appropriately early comprehensive interventions may be important in preventing deaths related to hypertension [32]. Unlike the population in the cited study, our analysis included apparently healthy individuals with no diagnosed risk factors or chronic diseases, which is an important aspect in the context of interpreting the compared data. It is worth recalling here the results of the GENVASC study in assessing the effectiveness of the NHS Health Check program. As mentioned earlier, the study included 27,888 participants, and a new diagnosis of hypertension was made in 2.3% of individuals. Comparing data from the general population without comorbidities can provide information on the effectiveness of screening in low-risk groups [26].

Unlike some large-scale screening programmes that have reported higher detection of previously undiagnosed hypertension, our study did not observe a clear difference between groups. However, this finding should be interpreted with caution. The small number of newly detected cases and the resulting limited statistical power lead to imprecise estimates and therefore preclude definitive conclusions regarding the absence of an effect. The observed pattern may reflect the relatively young study population and the widespread use of opportunistic blood pressure measurements in routine primary care, which could reduce the incremental yield of structured screening for hypertension in lower-risk populations.

### 4.4. Thyroid Diseases

The last diseases whose detectability was analyzed in the presented study were thyroid diseases: hypothyroidism and nodular goiter. The study showed statistically significant differences in the detection of new cases of the disease in the group of patients after the intervention. The available published scientific studies lack data directly assessing the effectiveness and possible benefits of screening for thyroid dysfunction. A review by the U.S. Preventive Services Task Force (USPSTF) published in 2015 showed that screening may increase the detection rate of thyroid diseases, but there is a lack of evidence on the benefits and risks that could result from the introduction of such screening. This highlights the need for further research to evaluate the effects of treatment in people with thyroid dysfunction detected during screening [33].

Direct comparison with other studies is limited, as evidence on population-based screening for thyroid dysfunction remains scarce and inconsistent. However, similar to previous reports, our findings suggest that structured assessments may increase detection of previously unrecognised thyroid abnormalities, while the clinical significance of such findings requires cautious interpretation.

### 4.5. Strengths and Limitations

The main limitation is differential diagnostic intensity (surveillance/ascertainment bias): BZ participants underwent systematic laboratory testing and structured assessment, whereas controls received usual care. This design increases the likelihood of detecting mild or subclinical abnormalities in the BZ group independently of true differences in disease occurrence. Therefore, findings should be interpreted as associations with diagnostic yield rather than causal effects on incidence or long-term outcomes.

This study was conducted in a single primary care practice and involved a locally adapted version of the Health Check-up tool. These features, together with the observational design and potential selection and detection biases, limit the generalisability of the findings to other settings or healthcare systems. In particular, differential diagnostic intensity between BZ participants and controls represents a major source of detection bias, as systematic testing in the BZ group may increase the likelihood of identifying subclinical or mild abnormalities independently of true disease prevalence. In this context, the interpretation of the present results should be aligned with the broader evidence based on preventive health checks.

The findings should also be interpreted in the context of the broader and heterogeneous evidence on general health checks and preventive screening programmes. While some studies have demonstrated increased detection of risk factors and diagnoses, evidence regarding long-term clinical benefits remains mixed. This underscores the need for more robust evaluations, including multi-centre studies and randomised designs, to better define the clinical and policy implications of structured preventive health checks.

Among the limitations of the study, it is worth noting the gender distribution in the study group, with women accounting for 60.2% of the population and men for 39.8%. This phenomenon may reflect greater health awareness and the tendency to take care of one’s health by participating in preventive programs among women. Similar conclusions were presented in a report by the National Institute of Public Health in a study titled The Health Situation of the Polish Population and its Determinants 2022. The report indicates that there is a noticeable difference in preventive behavior in Poland depending on gender, with women (51.3%) undergoing laboratory tests more often than men (40.2%) [34]. In addition, self-selection bias is an important factor and should be taken into account when assessing the effectiveness of voluntary interventions such as Health Checkups. Participants in preventive programs often differ significantly from the non-participating group in terms of socio-demographic characteristics, lifestyle, and overall health. They usually have a more favorable health profile, which may influence the results assessing the effectiveness of preventive programs [35].

Another limitation of the study relates to the age eligibility criteria of the POZ PLUS programme, which included adults aged 20–65 years. While current ESC guidelines recommend opportunistic cardiovascular risk screening starting from the age of 40 years without an upper age limit, the programme design was defined at the national level and could not be modified by individual healthcare providers. This discrepancy has been subject to professional debate in Poland, with some medical organisations questioning the clinical and economic justification of applying extensive laboratory testing across a broad age range. These considerations should be taken into account when interpreting the results and when designing future preventive programmes with more targeted, risk-based eligibility criteria [35].

An important limitation of this study is also the lack of systematically recorded data on key risk factors such as BMI, smoking status, and lifestyle variables. Their absence limited the possibility of statistical adjustment and increased the likelihood of residual confounding. Furthermore, this limitation is inherent to analyses based on routine primary care documentation rather than data collected specifically for research purposes.

Due to variability in individual entry times, detection measures quantify cumulative proportions of newly detected diagnoses over the study period, not incidence rates based on person-time at risk. Information on patient transfer out of the practice or death was not consistently available in the EHR, and therefore, formal censoring was not applied.

Such differences can introduce difficult-to-correct biases that significantly limit the ability to fully control the impact of external variables on detection rates and the validity of conclusions drawn from observational studies on the effectiveness of screening in the prevention of health events. Another potential limitation is the source of data, which is based on medical records from doctor’s visits, which carries the risk of omitting information that has not been properly documented and thus may not have been included in the analysis.

### 4.6. Implications for Practice and Future Research

The results obtained also prompt consideration of the validity of a more targeted approach to qualifying the population for screening. Critics of the program found no justification for performing all the laboratory tests that are necessary and essential for a basic check-up, i.e., complete blood count, ESR, blood glucose, lipid profile (including total cholesterol, LDL cholesterol, HDL cholesterol, and triglycerides), and urinalysis, on every patient participating in the program [36]. Health check-ups covered the general population, but focusing on high-risk groups could potentially increase the effectiveness and clinical relevance of interventions, although further research is needed to determine the optimal screening model.

The initial stages are often asymptomatic with a long preclinical phase. Early identification of these health conditions allows for earlier treatment, delays the progression of the disease, and may potentially reduce long-term healthcare costs, although further analysis and research in this area is needed [36].

Future research should focus on in-depth verification of the observed relationships in larger, more diverse populations and detailed analysis and assessment of the prevalence of risk factors associated with the occurrence of the analyzed disease entities in the study population. Further research should focus not only on the number of diagnoses, but also on the time it takes to diagnose and treatment outcomes. In addition, it seems reasonable to conduct studies that would assess the economic effectiveness of the analyzed preventive tool in the early detection of disease entities, which would allow for better targeting of health policy and preventive programs.

## 5. Conclusions

This study analysed the diagnostic yield of selected disease entities during the pilot Health Check-up programme in a primary care setting. Participation in the structured Health Check-up (BZ) was associated with higher recorded detection of lipid metabolism disorders, dysglycaemia, and thyroid conditions compared with usual care in this single-practice EHR-based analysis.

These findings indicate that structured preventive assessments in primary care may facilitate identification of selected metabolic and endocrine abnormalities. However, increased detection alone should not be interpreted as evidence of improved clinical effectiveness or patient outcomes.

In the context of the European experience, evidence from preventive programmes implemented in countries such as the United Kingdom, Italy, and Finland highlights the broader relevance of structured screening approaches in primary care while also underscoring the variability of their implementation and effects.

Future studies should assess whether increased diagnostic yield observed in structured Health Check-up programmes translates into improved treatment initiation, risk-factor control, patient-important outcomes, and cost-effectiveness, ideally in multi-centre studies with longer follow-up.

## Figures and Tables

**Table 1:** Characteristics of patients.

Characteristic	Intervention Group (BZ) (*n* = 865)	Control Group (*n* = 3199)
Sex, *n* (%)		
Female	521 (60.2%)	1577 (49.3%)
Male	344 (39.8%)	1622 (50.7%)
Age, years		
Average age	43.7	38.8
Median age	43.6	37.8

**Table 2:** Detection rate of selected disease entities.

ICD-10 Diagnosis	Intervention Group (BZ)				Control Group		
	n/N	(%)	[95% CI]	n/N	(%)	[95% CI]	*p*-Value
I10—Hypertension	40/865	4.62	3.3–6.3	144/3199	4.5	3.8–5.3	0.9505
E11—Type 2 diabetes	6/865	0.69	0.25–1.51	13/3199	0.41	0.22–0.69	0.4134
E78—Lipid disorders	89/865	10.29	8.26–12.66	83/3199	2.59	2.07–3.22	<0.001
R73—Abnormal fasting glucose	25/865	2.89	1.87–4.27	56/3199	1.75	1.32–2.27	0.0465
E03–E04—Thyroid diseases	37/865	4.27	3.01–5.9	72/3199	2.25	1.76–2.83	0.0016
More than 1 diagnosis	32/865	3.70	2.63–5.18	64/3199	2.00	1.57–2.55	0.00523

## Data Availability

Data are contained within the article.
